# Emerging Therapeutic Synergies: Combining PD-1 Inhibitors With Poly-ADP-Ribose Polymerase (PARP) Inhibitors in the Treatment of Gynecologic Cancers

**DOI:** 10.7759/cureus.99275

**Published:** 2025-12-15

**Authors:** Mohamed Sheeraz Mohamed Azhar, Zhiyu Loh, Francis T Mutamba, Ahmed Algahiny, Nafiu Mukhtar Yunusa, Sharwini Paramasevon, Manar Almusarhed

**Affiliations:** 1 Medical Oncology, University Hospitals of Leicester NHS Trust, Leicester, GBR; 2 Internal Medicine, University Hospitals of Leicester NHS Trust, Leicester, GBR; 3 Internal Medicine, University Hospitals Birmingham NHS Foundation Trust, Birmingham, GBR; 4 Internal Medicine, Kettering General Hospital NHS Foundation Trust, Kettering, GBR

**Keywords:** combination therapy, endometrial cancer, immunotherapy, ovarian cancer, parp inhibitor, pd-1/pd-l1

## Abstract

Gynecological cancers (ovarian, endometrial, cervical) remain a major cause of morbidity and mortality, driven by late presentation and resistance to standard therapies. Immune checkpoint blockade (PD-1/PD-L1) and PARP inhibition have each improved outcomes in biomarker-defined subsets. Preclinical data suggest synergy between these classes via PARP-induced DNA damage, cGAS-STING activation, and enhanced tumor immunogenicity, which may be amplified by PD-1/PD-L1 blockade. We performed a narrative review with a structured literature search of MEDLINE, Embase, and ClinicalTrials.gov (January 1, 2015, to August 24, 2025) for interventional trials evaluating a PARP inhibitor combined with an anti-PD-1/PD-L1 agent in ovarian, fallopian tube, primary peritoneal, endometrial, or cervical cancer. Eligibility required ≥1 efficacy endpoint (objective response rate {ORR}, progression-free survival {PFS}, and/or overall survival {OS}) plus safety in an extractable gynecology-only cohort (≥20 evaluable patients or any phase III). Triplets were eligible if the third agent was non-cytotoxic (e.g., bevacizumab). Regimens with concurrent cytotoxic chemotherapy in the investigational combination were excluded. Nine studies met the criteria (one phase III; eight phase I/II). In recurrent ovarian cancer, niraparib+pembrolizumab showed modest activity with durable responses in homologous recombination-deficient (HRD) tumors; olaparib+durvalumab demonstrated high activity in gBRCA platinum-sensitive relapse, and adding bevacizumab broadened benefit in non-BRCA cohorts. In the newly diagnosed disease, rucaparib+nivolumab maintenance failed to improve PFS versus rucaparib alone. Endometrial trials (olaparib+durvalumab; talazoparib+avelumab in mismatch repair-proficient disease) showed limited activity overall, with signals restricted to biomarker-selected subgroups. Toxicities reflected expected myelosuppression from PARP inhibitors and immune-related adverse events, generally manageable with standard algorithms. PARP+PD-1/PD-L1 combinations are most compelling in ovarian cancer, particularly in BRCA/HRD tumors and, in selected settings, with the addition of bevacizumab, while frontline maintenance benefit remains unproven and endometrial activity is modest. Biomarker-guided selection, rational triplets with non-cytotoxic partners, and optimized sequencing warrant further evaluation.

## Introduction and background

Gynecological cancers, including ovarian, endometrial, and cervical malignancies, remain a substantial global health burden. In 2023 and 2020, large epidemiological series confirmed that these tumors contribute significantly to cancer incidence and mortality worldwide, with regional variation in patterns of disease and outcomes [1-3]. Ovarian cancer, although representing a relatively small proportion of cancers in women, is the leading cause of gynecological cancer-related death due to late-stage presentation and high relapse rates [1-5]. Most women with advanced epithelial ovarian cancer experience disease recurrence following apparently successful first-line treatment, underscoring the need for more durable systemic therapies [4,5].

Endometrial cancer is now the most common gynecological malignancy in high-income countries [1,2]. Recent data show rising incidence across age groups and increasing mortality, particularly in older women, driven in part by obesity, metabolic risk factors, and biological heterogeneity [6-8]. Cervical cancer remains a leading cause of cancer death in women globally, especially in low- and middle-income countries where access to human papillomavirus (HPV) vaccination and organized screening remains limited [3,9].

The last decade has seen a paradigm shift in systemic therapy for gynecological cancers, driven by the introduction of targeted agents, particularly immune checkpoint inhibitors and poly-ADP-ribose polymerase (PARP) inhibitors. Immune checkpoint inhibitors targeting programmed cell death protein-1 (PD-1) and its ligand PD-L1 restore anti-tumor immune responses by reversing T-cell exhaustion [10,11]. PD-L1 expression has been documented across a wide range of solid tumors, including gynecological cancers, and correlates with distinct immune microenvironment patterns relevant to response to checkpoint blockade [10,11]. In endometrial cancer, PD-1 blockade combined with chemotherapy has improved outcomes, and pembrolizumab has demonstrated clinically meaningful activity in mismatch repair-deficient (dMMR) or microsatellite instability-high (MSI-H) solid tumors, including gynecological cancers [12,13]. These developments reflect a broader move towards immune-driven oncology in selected biomarker-defined populations.

In parallel, PARP inhibitors have transformed the management of epithelial ovarian cancer, particularly in patients with BRCA1/2 mutations or broader homologous recombination deficiency (HRD). By exploiting the principle of synthetic lethality, PARP inhibition leads to accumulation of unrepaired DNA damage and selective tumor cell death in HR-deficient tumors [14-16]. Pivotal trials of olaparib, niraparib, and rucaparib in both recurrent and first-line maintenance settings have demonstrated substantial improvements in progression-free survival, especially in BRCA-mutated and HRD-positive populations [17-23]. PARP inhibitors are now integral to standard-of-care maintenance strategies in advanced ovarian cancer. Preclinical data also suggest that PARP inhibitors can act synergistically with platinum-taxane chemotherapy, enabling dose reductions while maintaining cytotoxic efficacy [24].

Mounting preclinical and translational evidence suggests that combining PD-1/PD-L1 blockade with PARP inhibition may further enhance anti-tumor efficacy by leveraging complementary mechanisms [21,25-32]. PARP inhibition induces DNA damage and increases cytosolic DNA fragments, which activate the cyclic GMP-AMP synthase-stimulator of interferon genes (cGAS-STING) pathway, driving type I interferon production and recruitment of dendritic cells and CD8+ T cells [21,25-28,30]. PARP inhibitors also increase PD-L1 expression and augment neoantigen presentation, rendering tumors more immunogenic but also more dependent on PD-1/PD-L1-mediated immune escape [29,30]. PD-1/PD-L1 blockade can then restore T-cell effector function in this inflamed microenvironment, potentially converting immunologically “cold” tumors into “hot” ones [10,11,31,32].

These mechanistic insights have catalyzed a wave of preclinical and early-phase clinical studies exploring PD-1/PARP combinations in gynecological cancers. Immunogenomic profiling has begun to identify features associated with response to combined PARP and PD-1 inhibition, including HRD status, interferon signatures, and patterns of tumor-infiltrating lymphocytes [22,25]. At the same time, real-world data highlight ongoing unmet needs, particularly in older patients and those with relapsed disease, reinforcing the importance of more effective and tolerable treatment options [33,34].

Despite growing interest in combining PARP inhibitors with PD-1/PD-L1 blockade, the available evidence remains fragmented and dominated by small, early-phase studies. As a result, important uncertainties persist regarding optimal patient selection, sequencing, expected magnitude of benefit, and applicability across tumor subtypes. These gaps underscore the need for a structured synthesis of current clinical and mechanistic data.

Gynecological malignancies are biologically compelling candidates for PARP-PD-1 combination strategies. Ovarian cancers exhibit a high prevalence of homologous recombination defects; endometrial cancers with dMMR/MSI-H display strong immunogenicity and high neoantigen loads; and cervical cancers demonstrate virally driven immune activation. These biological attributes provide multiple convergent pathways through which PARP-induced DNA damage and immune checkpoint blockade may synergize.

Furthermore, recent developments in 2024-2025 have raised concerns regarding the long-term role of PARP inhibitor maintenance, particularly in homologous recombination-proficient disease. Issues include diminishing benefit over time, emerging resistance mechanisms, cumulative toxicity, and cost considerations. These debates have intensified interest in rational combination approaches capable of extending the therapeutic reach of PARP inhibitors. Taken together, these epidemiological, therapeutic, and mechanistic insights highlight both the promise and the complexity of integrating PARP inhibitors with PD-1/PD-L1 blockade in routine practice.

Objectives

This review synthesizes current evidence on PD-1/PD-L1 blockade combined with PARP inhibition in gynecological cancers. We outline the following four points in this study: (1) the underlying biological rationale for synergy, (2) the clinical efficacy and safety profile demonstrated in key trials, (3) biomarker-informed patient selection strategies, and (4) challenges and future directions for integrating these combinations into practice.

This structured narrative review aimed to clarify where evidence is strongest, where uncertainties remain, and how future research may refine the role of PD-1/PARP combinations in ovarian and endometrial cancers.

## Review

Methods

**Figure 1:**
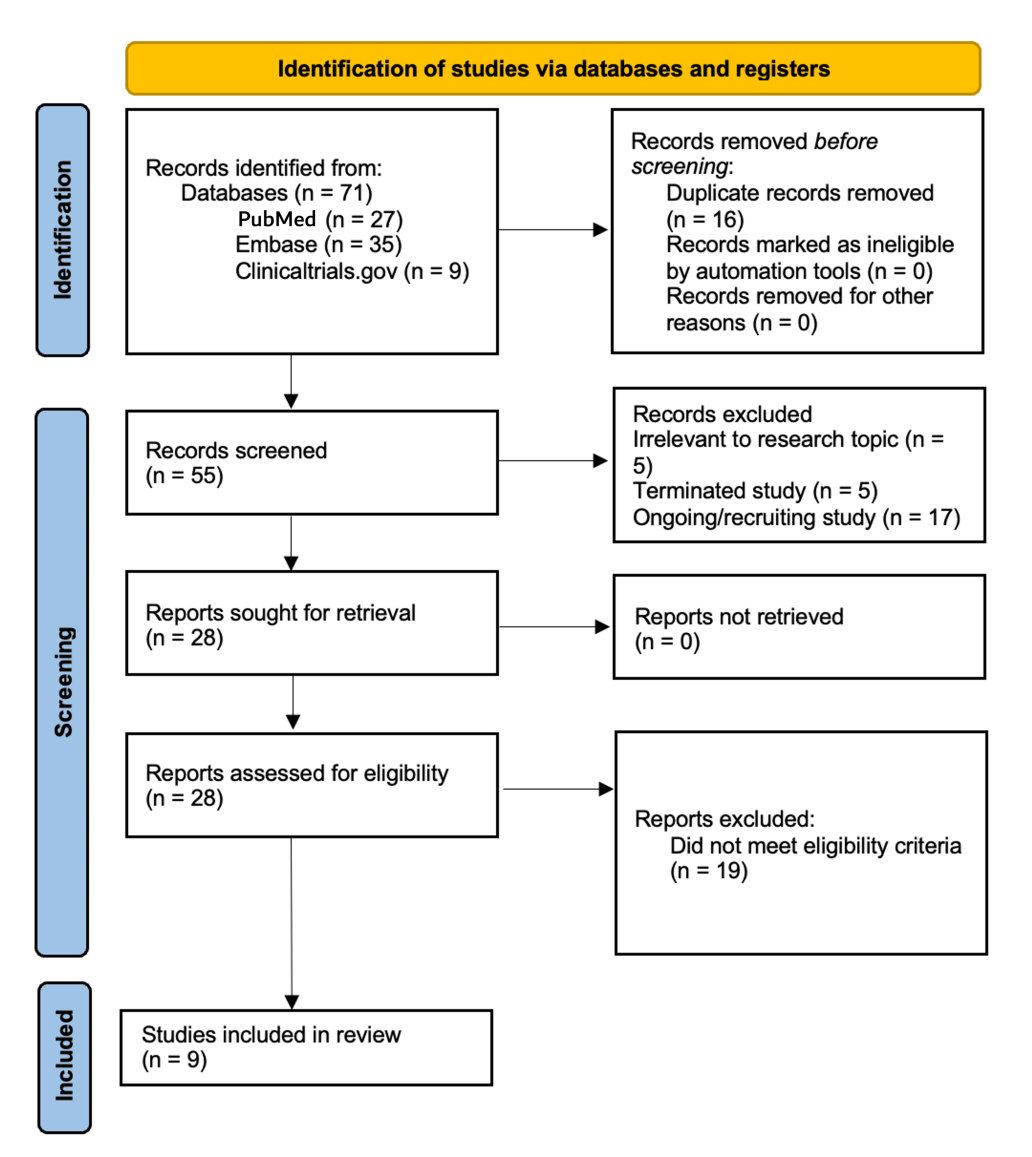
Flow diagram of study identification and selection for this narrative review of trials combining PARP inhibitors with PD-1/PD-L1 blockade in gynecological cancers. No formal risk-of-bias assessment was undertaken. PRISMA: Preferred Reporting Items for Systematic Reviews and Meta-Analyses

This review was conducted as a structured narrative synthesis. We systematically searched MEDLINE (PubMed), Embase, and ClinicalTrials.gov for interventional phase I-III clinical trials evaluating PARP inhibitors combined with PD-1/PD-L1 inhibitors in ovarian, fallopian tube, primary peritoneal, endometrial, or cervical cancer between January 1, 2015, and August 24, 2025. Data extraction included study design, eligibility criteria, sample size, interventions, biomarker-defined subgroups, objective response rate (ORR), progression-free survival (PFS), overall survival (OS), and safety outcomes, including rates of grade ≥3 adverse events. Mechanistic correlates were extracted where reported. The study selection process is summarized in Figure 1.

Because the majority of eligible studies were early-phase, single-arm, hypothesis-generating trials, formal risk-of-bias assessment using tools such as risk of bias 2 (ROB-2) or Newcastle-Ottawa was not appropriate and was therefore not performed. No review protocol was registered in PROSPERO. Although a structured search strategy and a Preferred Reporting Items for Systematic Reviews and Meta-Analyses (PRISMA)-style flow diagram are presented, the resulting analysis constitutes a narrative rather than a systematic review (Table 1).

**Table 1:** MEDLINE, Embase, and ClinicalTrials.gov search strategy for trials combining PARP inhibitors with PD-1/PD-L1 blockade in gynecological cancers.

Database/source	Platform/access	Search strategy used	Limits/filters	Date searched	Results retrieved
PubMed (MEDLINE)	PubMed.gov	((“ovarian cancer” OR “endometrial cancer” OR “cervical cancer” OR “gynecologic cancer”) AND (“PD-1” OR “PD-L1” OR “immune checkpoint inhibitor” OR pembrolizumab OR nivolumab OR durvalumab OR dostarlimab) AND (“PARP inhibitor” OR olaparib OR niraparib OR rucaparib OR talazoparib OR veliparib))	Humans, English	January 1, 2015, to August 24, 2025	27
Embase	Embase via Ovid/SP	1. exp ovary cancer/ 2. exp endometrium cancer/ 3. exp uterine cervix cancer/ 4. exp female genital tract cancer/ 5. 1 or 2 or 3 or 4:ti,ab.mp. 6. pd-1 receptor.mp. 7. pd-l1.mp. 8. exp immune checkpoint inhibitor/ 9. exp pembrolizumab/ 10. exp nivolumab/ 11. exp durvalumab/ 12. exp dostarlimab/ 13. 6 or 7 or 8 or 9 or 10 or 11 or 12:ti,ab.mp. 14. exp nicotinamide adenine dinucleotide adenosine diphosphate ribosyltransferase inhibitor/ 15. exp olaparib/ 16. exp niraparib/ 17. exp rucaparib/ 18. exp talazoparib/ 19. exp veliparib/ 20. 14 or 15 or 16 or 17 or 18 or 19:ti,ab.mp. 21. 5 and 13 and 20	Clinical trial, human, English, female	January 1, 2015, to August 24, 2025	35
ClinicalTrials.gov	ClinicalTrials.gov advanced search	(“ovarian cancer” OR “endometrial cancer” OR “cervical cancer” OR “gynecologic cancer”) AND (“PD-1” OR “PD-L1” OR “immune checkpoint inhibitor” OR pembrolizumab OR nivolumab OR durvalumab OR dostarlimab) AND (“PARP inhibitor” OR olaparib OR niraparib OR rucaparib OR talazoparib OR veliparib)	Active and completed studies; phase: 1, 2, 3; interventional studies; studies with results	January 1, 2015, to August 24, 2025	9

Combinations containing cytotoxic chemotherapy were excluded because overlapping myelosuppression could obscure the biological and immunological effects attributable to PARP inhibition, thereby confounding evaluation of PARP-PD-1 synergy. Only non-cytotoxic triplet combinations (e.g., bevacizumab-containing regimens) were included. Where relevant, evidence strength was weighted according to study phase, with phase III randomized data prioritized over single-arm or preliminary phase I/II cohorts.

Biological rationale for PD-1/PARP combinations

PARP enzymes play a central role in DNA single-strand break repair via base excision repair. Inhibition of PARP leads to the accumulation of single-strand breaks that are converted to double-strand breaks during replication. In cells proficient in homologous recombination (HR), these lesions are repaired via BRCA1/2-mediated pathways; in HR-deficient cells, including many BRCA-mutated or HRD-positive ovarian cancers, they result in cell death through synthetic lethality [14-16]. In addition, PARP inhibitors can “trap” PARP-DNA complexes on chromatin, further disrupting replication and increasing cytotoxicity [23]. Preclinical data also suggest that PARP inhibitors can act synergistically with platinum-taxane chemotherapy, enabling dose reductions while maintaining cytotoxic efficacy [24].

Beyond direct cytotoxicity, PARP inhibition has key immunomodulatory effects. DNA damage caused by PARP inhibitors generates cytosolic DNA fragments that can activate the cGAS-STING pathway, driving type I interferon production and upregulating interferon-stimulated genes [21,25-28]. Preclinical models in ovarian, breast, and lung cancer have shown that PARP inhibition can increase tumor mutational burden, enhance antigen presentation, and upregulate PD-L1 expression, collectively creating a more inflamed tumor microenvironment [21,25-30]. PARP inhibition has also been associated with increased CD8+ T cell infiltration and a shift towards an immune-active phenotype in HR-deficient tumors [21,22,25-28].

Immune checkpoint inhibitors targeting PD-1/PD-L1 act by reversing T cell exhaustion and restoring cytotoxic function [10,11,35]. In endometrial and cervical cancers, PD-1 blockade has been particularly effective in tumors with dMMR/MSI-H or high PD-L1 expression, reflecting their immunogenic biology [8,9,12,13]. The convergence of HRD-associated genomic instability, PARP-induced cGAS-STING activation, and PD-L1 upregulation provides a strong rationale for combining PARP inhibitors with PD-1/PD-L1 blockade in gynecological cancers [21,22,25-32].

Clinical trial evidence

A number of early-phase and phase III trials have evaluated combinations of PARP inhibitors with PD-1 or PD-L1 blockade across ovarian and endometrial cancer. These studies vary considerably in design, biomarker selection, and clinical context, resulting in heterogeneous outcomes. The following sections summarize the major efficacy and safety findings from these key trials. The key characteristics and outcomes of the included clinical trials are summarized in Table 2.

**Table 2:** Summary of key PARP-PD-1/PD-L1 combination trials in gynecological cancers. DCR: disease control rate; pMMR: proficient mismatch repair; HRR: homologous recombination repair; OC: ovarian cancer; EC: endometrial cancer; PROC: platinum-resistant ovarian cancer; PR: platinum-resistant; PS: platinum-sensitive; ORR: overall response rate; PFS: progression-free survival; OS: overall survival; HR: hazard ratio; LFTs: liver function tests; AE: adverse event

Trial (phase), studies	Population	Intervention	Key efficacy outcomes	Notable adverse events	Key takeaway
TOPACIO/KEYNOTE-162 (I/II), Konstantinopoulos et al. (2019) [36]	Recurrent PROC (BRCA^+^ and BRCA^-^)	Niraparib+pembrolizumab	ORR 18%, DCR 65%; benefit seen regardless of BRCA status	Anemia (21%), thrombocytopenia (9%)	Activity across biomarker groups; HRD-positive patients showed higher response
ATHENA-COMBO (III), Monk et al. (2024) [37]	Newly diagnosed advanced OC post-platinum response	Rucaparib+nivolumab versus rucaparib	PFS: 15.0 versus 20.2 months (HR: 1.3); no statistical benefit	Anemia (27%), neutropenia (25%), deranged LFTs (21%)	No added benefit in frontline maintenance
Lampert et al. (2020) (II) [38]	Recurrent OC; mostly PR	Olaparib+durvalumab	ORR: 14%, DCR: 71%; responses seen regardless of BRCA status	Anemia (31%), lymphopenia (20%)	Missed primary endpoint. May benefit biomarker-unselected patients
MEDIOLA (II), Drew et al. (2024) [39]	PS recurrent OC, gBRCA^+^, and non-BRCA	Olaparib+durvalumab±bevacizumab	gBRCA^+^: ORR 92%, PFS: 15 months; non-BRCA triplet: DCR: 74%, OS: 31.9 months	Anemia, neutropenia	High activity in BRCA^+^; triplet may benefit non-BRCA
Konstantinopoulos et al. (2022) (II) [40]	Recurrent pMMR EC	Talazoparib+avelumab	ORR: 11%, six-month PFS: 23%	Anemia (46%), thrombocytopenia (29%), neutropenia (11%)	Meets primary endpoint; most benefits in patients with HRR alterations
DOMEC (II), Post et al. (2022) [41]	Advanced or recurrent EC	Olaparib+durvalumab	Six months PFS rate 34%, median OS 8.4 months, ORR: 16%	Anemia (10%); dose reduction in 24%	Did not meet primary endpoint; potential benefit in germline BRCA1 mutation
GINECO-BOLD (II), Freyer et al. (2024) [42]	Relapsed high-grade OC (PR versus PS)	Durvalumab+olaparib+bevacizumab	PR: three-month non-progression rate: 70% PS: six-month non-progression rate: 44%	Consistent with known toxicities	Activity of triplet in PR cohort
OPEB-01 (II), Kim et al. (2023) [43]	BRCA-wt, PS recurrent OC	Olaparib+pembrolizumab+bevacizumab	Six months PFS rate: 89%, median PFS: 22 months	Anemia (23%); dose reductions/interruptions are frequent	Efficacy in BRCA-wt PS recurrent OC
OPAL (II), Liu et al. (2024) [44]	Advanced PROC	Niraparib+dostarlimab+bevacizumab	ORR: 17.1%; DCR: 73.2%	High toxicity rate (92.7% grade ≥3 adverse events); hypertension (26.8%)	Modest activity, significant toxicity

Doublet regimens in ovarian cancer

*TOPACIO/KEYNOTE-162 (Niraparib+Pembrolizumab; Phase I/II)*​​​​

TOPACIO/KEYNOTE-162 was an open-label, non-randomized phase I/II study evaluating niraparib plus pembrolizumab in recurrent, platinum-resistant or refractory ovarian carcinoma [36]. Sixty-two patients were enrolled, of whom 60 were evaluable for efficacy. The median age was approximately 60 years, and patients were heavily pre-treated (median of three prior lines, up to five). Both BRCA-mutated and BRCA-wild-type tumors were included, representing a heterogeneous, treatment-resistant population.

The ORR was 18% overall, with similar response rates in BRCA-mutated (18%) and BRCA-wild-type (19%) tumors. The disease control rate (DCR) was 65%, and responses were observed regardless of platinum sensitivity. Median duration of response was 9.3 months, although median PFS remained modest at 3.4 months (95% CI, 2.1-5.1). Responses were more frequent in HRD-positive tumors, aligning with the mechanistic rationale for this combination [14-16,25-28].

Treatment was generally well tolerated. The most common grade ≥3 toxicities were anemia (21%) and thrombocytopenia (9%), consistent with prior niraparib experience [18,22]. Non-hematological adverse events (fatigue, nausea, decreased appetite) were predominantly grade 1-2. As a small, single-arm study, TOPACIO is best viewed as a signal-generating phase I/II trial, showing that PARP-PD-1 combinations can achieve durable responses in selected, heavily pre-treated patients, but without providing definitive comparative evidence.

ATHENA-COMBO (Rucaparib+Nivolumab Versus Rucaparib; Phase III)

ATHENA-COMBO was the first large phase III trial to test PARP-PD-1 doublet maintenance in newly diagnosed ovarian cancer [37]. Patients with International Federation of Gynecology and Obstetrics (FIGO) stage III-IV high-grade ovarian cancer who had responded to first-line platinum-based chemotherapy were randomized 1:1 to rucaparib plus nivolumab or rucaparib alone.

In the intention-to-treat population, the doublet failed to improve PFS as follows: median PFS was 15.0 months with rucaparib+nivolumab versus 20.2 months with rucaparib monotherapy (HR: 1.3; 95% CI: 1.1-1.5). Subgroup analyses based on HRD status and PD-L1 expression did not show an advantage for the combination. OS data were immature at the time of reporting. Toxicities were consistent with known PARP-related adverse events but more frequent in the combination arm. Grade ≥3 anemia (27%), neutropenia (25%), and elevated transaminases (21%) were more common with rucaparib+nivolumab than rucaparib alone. Immune-related toxicities were not markedly increased. Importantly, as a phase III randomized trial, ATHENA-COMBO provides the strongest available evidence and suggests that adding nivolumab to rucaparib in an unselected frontline maintenance population does not improve outcomes and may worsen PFS. This contrasts with the more optimistic signal from phase I/II trials and emphasizes the importance of disease setting and biomarker selection.

Olaparib+Durvalumab in Recurrent Ovarian Cancer (Lampert et al.; Phase II)

Lampert et al. conducted a single-arm, single-center phase II study of olaparib plus durvalumab in recurrent ovarian cancer, focusing on a cohort of 35 patients, predominantly with platinum-resistant disease [38]. The study did not meet its primary ORR endpoint, achieving an ORR of 14% (95% CI: 4.8-30.3%). Responses occurred across biomarker strata, including both BRCA-mutated and BRCA-wild-type tumors, and the DCR was 71% [40].

The combination was generally well tolerated. The most common grade ≥3 treatment-related adverse events were anemia (31%) and lymphopenia (20%), in keeping with the expected profile of olaparib [6,11,31-34,40]. Although the primary endpoint was not met, the durability of disease control in a heavily pre-treated population suggested a signal of benefit in selected patients. As with TOPACIO, this phase II, single-arm study supports the presence of a clinical signal but cannot establish superiority over PARP monotherapy or alternative regimens.

MEDIOLA (Olaparib+Durvalumab±Bevacizumab; Phase II)

MEDIOLA was a multi-cohort phase II trial assessing olaparib plus durvalumab, with or without bevacizumab, in PARP inhibitor-naïve, platinum-sensitive relapsed ovarian cancer [39]. Fifty-one patients were enrolled in the germline BRCA-mutated (gBRCA) cohort and 63 in non-BRCA cohorts (32 doublet; 31 triplet).

In the gBRCA cohort receiving olaparib+durvalumab, the confirmed ORR was 92.2% (95% CI: 81-98), with a median PFS of 15.0 months (95% CI: 12.9-24.1) and a median duration of response of 14.8 months. At 24 months, 76.7% of patients were alive. These results were consistent with the high sensitivity of gBRCA-mutated platinum-sensitive ovarian cancer to PARP-based strategies [14-20]. By contrast, in non-BRCA patients, the doublet achieved a 24-week DCR of only 28%. Addition of bevacizumab to create a triplet regimen increased the 24-week DCR to 74%, extended median PFS to 14.7 months, and improved OS to 31.9 months versus 26.1 months with the doublet. This suggests that anti-angiogenic therapy may augment PARP-PD-L1 combinations in biomarker-negative populations by improving immune-cell infiltration and drug delivery [31,32].

Across cohorts, grade ≥3 adverse events (AEs) occurred in 47-66% of patients, most commonly anemia and neutropenia. The triplet did not introduce unexpected toxicities, and treatment-related discontinuations were infrequent. MEDIOLA, although limited by its non-randomized phase II design, highlights the following two key points: (1) doublet PARP-PD-L1 therapy is highly active in gBRCA-mutated, platinum-sensitive disease; and (2) triplet regimens with bevacizumab may partially compensate for the absence of BRCA mutations.

PARP-PD-1 doublets in endometrial cancer

Endometrial cancer offers a distinct biological context, with dMMR/MSI-H tumors showing marked sensitivity to PD-1 blockade alone [8,12,13]. Two key trials have evaluated PARP-PD-1/PD-L1 combinations in largely proficient mismatch repair (pMMR) or molecularly unselected populations.

Konstantinopoulos et al. (Talazoparib+Avelumab; Phase II)

In a phase II trial, talazoparib plus avelumab was assessed in 35 patients with recurrent pMMR endometrial cancer [40]. The ORR was modest at 11.4% (all partial responses), but the trial met its co-primary endpoint with a six-month PFS rate of 22.9%. Median PFS was 3.6 months. Exploratory analyses suggested that patients with homologous recombination repair (HRR) gene alterations or a longer platinum-free interval (PFI ≥6 months) were more likely to benefit.

Hematological toxicities predominated, with grade ≥3 anemia (46%), thrombocytopenia (29%), and neutropenia (11%) being the most frequent treatment-related AEs. The pronounced hematologic toxicity observed with talazoparib-containing combinations is consistent with talazoparib’s uniquely high PARP-trapping potency [23]. Compared with olaparib or rucaparib, talazoparib forms more stable PARP-DNA complexes, causing replication-fork stalling and extensive DNA break accumulation not only in tumor cells but also in hematopoietic progenitors [23]. This mechanistic feature explains the higher rates of grade ≥3 anemia and thrombocytopenia reported across talazoparib studies. No patients discontinued therapy due to toxicity. This single-arm phase II trial, therefore, suggests modest activity in a difficult-to-treat, biomarker-restricted population, with potential enrichment in HRR-altered tumors.

When viewed alongside the efficacy of PD-1 monotherapy in biomarker-selected disease, the modest activity of talazoparib + avelumab becomes more apparent. In MSI-H/dMMR endometrial tumors, PD-1 inhibitors such as pembrolizumab achieve ORRs of 40-45% with durable disease control [12,13], far exceeding the outcomes seen in these predominantly pMMR populations. This contrast underscores the biological challenge of using PARP-ICI combinations in tumors lacking intrinsic immunogenicity or HRD-associated genomic instability.

DOMEC (Durvalumab+Olaparib; Phase II)

The DOMEC trial was a single-arm, multicenter phase II study evaluating durvalumab plus olaparib in 50 evaluable patients with advanced or recurrent endometrial cancer, largely unselected for molecular subtype [41]. Most patients (84%) had received prior chemotherapy. The primary endpoint, a six-month PFS rate of 50%, was not met; the observed six-month PFS rate was 34%. Median PFS was 3.4 months and median OS was 8.4 months; ORR was 16%. Notably, all three patients with known germline BRCA1 mutations achieved objective responses, indicating that this subgroup may derive particular benefit. The combination was well tolerated. Grade 3 treatment-related AEs were seen in 16% of patients, with anemia (10%) the most common. No grade 4 or 5 treatment-related events occurred. Dose reductions of olaparib were required in 24% of patients, and treatment discontinuation due to toxicity was rare (4%).

As with talazoparib+avelumab, the DOMEC results highlight the difficulty of generating meaningful benefit in unselected or pMMR endometrial cancer, particularly when PD-1 monotherapy already achieves high response rates in MSI-H/dMMR disease. Together, these findings reinforce the need for biomarker-driven patient selection and suggest that PARP-ICI combinations may hold value only in genomically defined subgroups, such as BRCA-mutated tumors.

Triplet regimens with bevacizumab

Triplet regimens incorporating an anti-angiogenic agent have been explored to further enhance PARP-PD-1/PD-L1 efficacy, based on the ability of vascular endothelial growth factor (VEGF) blockade to normalize vasculature, improve immune-cell trafficking, and modulate the tumor microenvironment [31,32].

GINECO-BOLD (Durvalumab+Olaparib+Bevacizumab; Phase II)

GINECO-BOLD was an open-label, multicenter phase II trial evaluating bevacizumab, olaparib, and durvalumab in relapsed high-grade ovarian cancer, with separate cohorts for platinum-resistant (n=41) and platinum-sensitive (n=33) disease [42]. In the platinum-resistant cohort, the three-month non-progression rate was 69.8%, meeting the primary endpoint; median PFS was 4.1 months. In the platinum-sensitive cohort, the six-month non-progression rate was 43.8%, which did not meet the predefined threshold; median PFS was 4.9 months. Overall, the triplet showed promising activity in heavily pre-treated, platinum-resistant patients but less clear benefit in the platinum-sensitive setting. The safety profile was manageable and consistent with the known toxicities of the individual agents, with no new safety signals or toxic deaths.

OPEB-01/APGOT-OV4 (Olaparib+Pembrolizumab+Bevacizumab; Phase II)

OPEB-01/APGOT-OV4 was a single-arm, multicenter phase II trial of triplet maintenance therapy with olaparib, pembrolizumab, and bevacizumab in 44 women with BRCA-wild-type, platinum-sensitive recurrent ovarian cancer who had achieved a response or stable disease after platinum-based chemotherapy. The trial met its primary endpoint, with a six-month PFS rate of 88.6%. Median PFS was 22.4 months, and median OS was 28.6 months, suggesting substantial and durable benefit in this biomarker-negative but chemo-sensitive population. However, toxicity was considerable: 52.3% of patients experienced grade ≥3 AEs, most commonly anemia (22.7%). Dose reductions of olaparib (61.4%) and dose interruptions of any study drug (86.4%) were frequent. One patient developed grade 4 myelodysplastic syndrome. Despite this, treatment discontinuation due to toxicity was rare, and the authors concluded that the regimen was manageable with active monitoring.

OPAL (Niraparib+Dostarlimab+Bevacizumab; Phase II)

The OPAL phase II trial evaluated niraparib, dostarlimab, and bevacizumab in pre-treated, advanced platinum-resistant ovarian cancer. Cohort A enrolled 41 patients. The triplet achieved an ORR of 17.1% and a DCR of 73.2%, indicating modest antitumor activity in this difficult-to-treat setting. Responses were observed regardless of BRCA, HRD, or PD-L1 status and occurred mainly in bevacizumab-naïve patients. Toxicity was substantial as follows: 92.7% of patients experienced grade ≥3 treatment-emergent AEs, with hypertension (26.8%) the most common, reflecting the bevacizumab component. The regimen, therefore, raised concerns about tolerability, particularly in the heavily pre-treated, platinum-resistant population.

In aggregate, triplet data indicate that bevacizumab-containing regimens can deliver impressive and durable PFS in selected settings (notably BRCA-wild-type, platinum-sensitive recurrence as in OPEB-01) but at the cost of substantial toxicity. In more heavily pre-treated, platinum-resistant disease (GINECO-BOLD resistant cohort, OPAL), activity is more modest and must be weighed carefully against tolerability.

Biomarkers and patient selection

Across these trials, the clinical efficacy of PARP-PD-1/PD-L1 combinations appears highly dependent on the underlying molecular and immune context of the tumor. The most established predictors are those linked to PARP inhibitor sensitivity. BRCA1/2 mutations, both germline and somatic, remain the best-validated biomarkers of PARP inhibitor benefit in ovarian cancer [14-20]. The exceptional ORR of 92.2% in the gBRCA-mutated cohort of MEDIOLA exemplifies this [39].

HRD, encompassing BRCA and non-BRCA defects in homologous recombination, is also an important determinant of PARP sensitivity, captured through genomic scar signatures and gene panels [5,14-16]. TOPACIO suggested higher and more durable responses in HRD-positive tumors [36], and preclinical data support a closer interplay between HRD, DNA damage, and immune activation [14-16,21,22,25-30]. However, ATHENA-COMBO did not identify HRD or PD-L1 as predictive of benefit from adding nivolumab to rucaparib in frontline maintenance, highlighting that biomarker effects are context-dependent [37].

Immunotherapy biomarkers show tumor-specific patterns. In ovarian cancer, PD-L1 expression and tumor-infiltrating lymphocytes have not consistently predicted benefit from PD-1 blockade, and single-agent PD-1 activity has been modest [10,11,31,35,45]. For endometrial cancer, MSI-H and dMMR clearly identify patients who benefit from PD-1 inhibition [8,12,13]. In the PARP-ICI setting, DOMEC’s lack of benefit in molecularly unselected endometrial cancer contrasts with the activity observed in BRCA-mutated patients and in pMMR tumors harboring HRR alterations in the talazoparib-avelumab trial, pointing towards a convergence of DDR and immune biomarkers [40,41].

The differential performance of PARP-PD-1 combinations across tumor subtypes can be explained mechanistically. BRCA-mutated and HRD-positive tumors accumulate unrepaired DNA breaks, activate cGAS-STING signaling, upregulate PD-L1, generate neoantigens, and exhibit inflamed microenvironments - all features that favor synergy with PD-1 blockade. Platinum sensitivity further correlates with ongoing dependency on homologous recombination repair. In contrast, HR-proficient and platinum-resistant tumors display lower genomic instability, weaker innate immune activation, reduced T-cell infiltration, and more pronounced immunosuppressive signaling, thereby limiting the biological conditions necessary for meaningful synergy.

Collectively, these biomarker patterns underline the importance of biologically driven patient selection when considering PARP-PD-1 combinations. To aid interpretation, Table 3 summarizes the principal biomarkers of interest across the main gynecological tumor types.

**Table 3:** Summary of biomarker relevance for PARP-PD-1/PD-L1 combinations across gynecological cancers. HPV: human papillomavirus

Tumor type	PARP-related biomarkers (PARP backbone)	Immunotherapy biomarkers (PD-1/PD-L1)	Combination-relevant comments
Ovarian cancer	Germline/somatic BRCA1/2; HRD scores; HRR gene alterations	PD-L1 expression (inconsistent); TILs; interferon-gamma signatures	Strongest data in BRCA/HRD-positive, platinum-sensitive disease (e.g. MEDIOLA); frontline unselected maintenance negative (ATHENA-COMBO).
Endometrial cancer	BRCA1/2 (rare); broader HRR alterations; HRD (exploratory)	MSI-H/dMMR (established), PD-L1 expression	PARP-ICI activity is modest overall; signals in HRR/BRCA-altered pMMR tumors; MSI-H/dMMR tumors may do well with ICI alone.
Cervical cancer	Limited data; PARP biomarkers are largely unexplored clinically	PD-L1 expression (KEYNOTE-158); HPV-related immunogenicity	No mature PARP-ICI combination data meeting inclusion criteria; any future trials likely to prioritize PD-L1 and viral/immune signatures.

In the future, integrated biomarker strategies will likely be required, combining BRCA/HRD status, HRR gene alterations, MSI-H/dMMR status, PD-L1 expression, interferon-gamma signatures, and broader immune microenvironment features [14-16,22,25,31,32]. Dynamic tools, such as circulating tumor DNA, exosomal RNA, and serial immune profiling, may help track response and emerging resistance in real time.

Challenges: resistance, toxicity, and access

Mechanisms of resistance represent a major barrier. For PARP inhibitors, resistance can develop through secondary “reversion” mutations in BRCA1/2 that restore HRR, loss of PARP1, upregulation of alternative repair pathways, or stabilization of replication forks [5,14-16]. For immune checkpoint blockade, resistance may arise from loss of antigen presentation, altered interferon signaling, recruitment of immunosuppressive myeloid or regulatory cell populations, and upregulation of alternative inhibitory receptors, such as TIM-3 and LAG-3 [10,31]. When PARP inhibitors and ICIs are combined, these resistance mechanisms can intersect in complex ways; for example, chronic STING activation may ultimately promote T-cell exhaustion and inflammatory tolerance [22,31,32].

Toxicity is another key challenge. PARP inhibitors are associated with myelosuppression (anemia, thrombocytopenia, neutropenia), fatigue, and gastrointestinal symptoms [14-20,23]. ICIs carry the risk of immune-related adverse events affecting multiple organ systems [10,11]. Combination regimens often yield additive toxicity, as seen in the high rates of grade ≥3 AEs in TOPACIO, MEDIOLA, OPEB-01, GINECO-BOLD, and OPAL [36-44]. Effective management requires proactive monitoring, early recognition and treatment of immune-related toxicities (often with corticosteroids), and judicious dose interruptions or reductions of PARP inhibitors.

Lastly, cost and access pose significant barriers. Both PARP inhibitors and ICIs are high-cost therapies, and access to HRD and MSI-H/dMMR testing is variable, particularly in resource-constrained settings [5, 8,32-34]. Without such biomarker testing, rational patient selection is impossible, risking both over- and under-treatment. Addressing these issues will require coordinated health system strategies, value-based pricing, and investment in diagnostic infrastructure.

Future directions

To contextualize future research priorities, it is important to consider the relative strength of the available evidence. Most encouraging results supporting PARP-PD-1/PD-L1 combinations come from small, early-phase (phase I/II) single-arm studies such as MEDIOLA, TOPACIO, OPEB-01, and the talazoparib-avelumab trial, which are hypothesis-generating but not definitive. These studies often include heterogeneous, heavily pre-treated populations and lack comparator arms, limiting the interpretation of clinical benefit. In contrast, the only phase III randomized evidence to date - ATHENA-COMBO - did not demonstrate added benefit from PD-1 inhibition when combined with a PARP inhibitor in the frontline maintenance setting. Similarly, neutral findings from DOMEC in unselected endometrial cancer further highlight that early activity signals do not necessarily translate into meaningful benefit at scale. Overall, the current evidence base remains preliminary, with biological plausibility and early-phase activity supporting further investigation, but without robust phase III confirmation. Future research should therefore prioritize adequately powered randomized trials, biomarker-enriched cohorts, and careful stratification by disease subtype and treatment setting.

Beyond the trials included in our formal data extraction, several large phase III studies that incorporate PARP inhibitors and PD-1/PD-L1 blockade alongside platinum-based chemotherapy provide important contextual information. In endometrial cancer, the DUO-E trial randomized patients with newly diagnosed advanced or recurrent disease to chemotherapy alone, chemotherapy plus durvalumab followed by durvalumab maintenance, or chemotherapy plus durvalumab followed by durvalumab and olaparib maintenance. Durvalumab-containing regimens significantly improved progression-free survival, with the greatest benefit seen when olaparib was added in mismatch repair-proficient tumors, leading to regulatory approval of durvalumab-olaparib combinations for pMMR advanced or recurrent endometrial cancer in 2024 [46]. In ovarian cancer, the FIRST/ENGOT-OV44 trial evaluated the addition of dostarlimab to first-line platinum-based chemotherapy followed by niraparib maintenance, with or without bevacizumab. Although the study met its primary endpoint, showing a statistically significant progression-free survival advantage for the dostarlimab-niraparib arm, the absolute benefit was modest (approximately 1-1.5 months), and no overall survival improvement has been demonstrated to date [47]. Together with the negative ATHENA-COMBO results, these trials suggest that simply layering PD-1/PD-L1 blockade onto a PARP inhibitor backbone and standard chemotherapy does not guarantee clinically meaningful benefit, underscoring the need for refined biomarker-driven designs [37,46,47].

## Conclusions

The convergence of PD-1 immune checkpoint inhibition and PARP-mediated DNA damage repair blockade represents one of the most promising emerging strategies in gynecological oncology. This approach exploits the following two complementary mechanisms: PARP inhibitors create DNA damage and activate innate immune sensing via cGAS-STING, while PD-1/PD-L1 blockade restores T cell-mediated cytotoxicity in an increasingly inflamed tumor microenvironment. Preclinical work has consistently demonstrated synergy, and early-phase clinical trials such as TOPACIO/KEYNOTE-162 and MEDIOLA have confirmed meaningful activity, particularly in BRCA-mutated and HRD-positive ovarian cancer. Triplet regimens incorporating bevacizumab have further extended benefit to selected BRCA-wild-type, platinum-sensitive populations, albeit with increased toxicity. By contrast, the only phase III trial to date, ATHENA-COMBO, did not show benefit for adding nivolumab to PARP inhibitor maintenance in unselected frontline ovarian cancer, and phase II data in largely pMMR or unselected endometrial cancer have been modest, with benefit confined to biomarker-enriched subgroups.

These findings underline that the strength of evidence remains limited, being dominated by small, non-randomized phase I/II studies with heterogeneous populations and endpoints, and a single negative phase III trial in the frontline setting. In addition, this work is a narrative review with a structured search, without formal risk-of-bias assessment or protocol registration, and therefore cannot claim the methodological robustness of a full systematic review. As such, the conclusions should be regarded as hypothesis-generating rather than definitive practice-changing guidance. Recent phase III trials, including DUO-E in endometrial cancer and FIRST/ENGOT-OV44 in ovarian cancer, illustrate that PARP-PD-1 combinations can yield statistically significant progression-free survival gains in selected settings, but that absolute benefits may be modest and context-dependent when added to intensive chemotherapy backbones. Progress is further constrained by the emergence of resistance, overlapping toxicities, and inequities in access to both drugs and companion diagnostics. Addressing these challenges will require refined biomarker strategies, rational triplet or multimodal regimens tailored to tumor biology, and optimized sequencing and duration of therapy. If these hurdles can be overcome, PD-1/PARP combinations may evolve from a promising niche approach to a targeted standard of care for biomarker-selected patients with advanced gynecological cancers, improving long-term survival and bringing the field closer to truly personalized, immune-driven oncology for women worldwide.
